# 5-(2,6-Difluoro­phen­yl)-1,3,4-thia­diazol-2-amine

**DOI:** 10.1107/S1600536809047990

**Published:** 2009-11-18

**Authors:** Yao Wang, Rong Wan, Feng Han, Peng Wang

**Affiliations:** a Department of Applied Chemistry, College of Science, Nanjing University of Technology, No. 5 Xinmofan Road, Nanjing, Nanjing 210009, People’s Republic of China

## Abstract

The title compound, C_8_H_5_F_2_N_3_S, was synthesized by the reaction of 2,6-difluoro­benzoic acid and thio­semicarbazide. The dihedral angle between the thia­diazole and phenyl ring is 35.19 (14)°. In the crystal structure, inter­molecular N—H⋯N hydrogen bonds form chains along the *b* and *c* axes.

## Related literature

For the biological activity of 1,3,4-thia­diazole derivatives, see: Nakagawa *et al.* (1996 ▶); Wang *et al.* (1999 ▶). For bond-length data see: Allen *et al.* (1987 ▶).
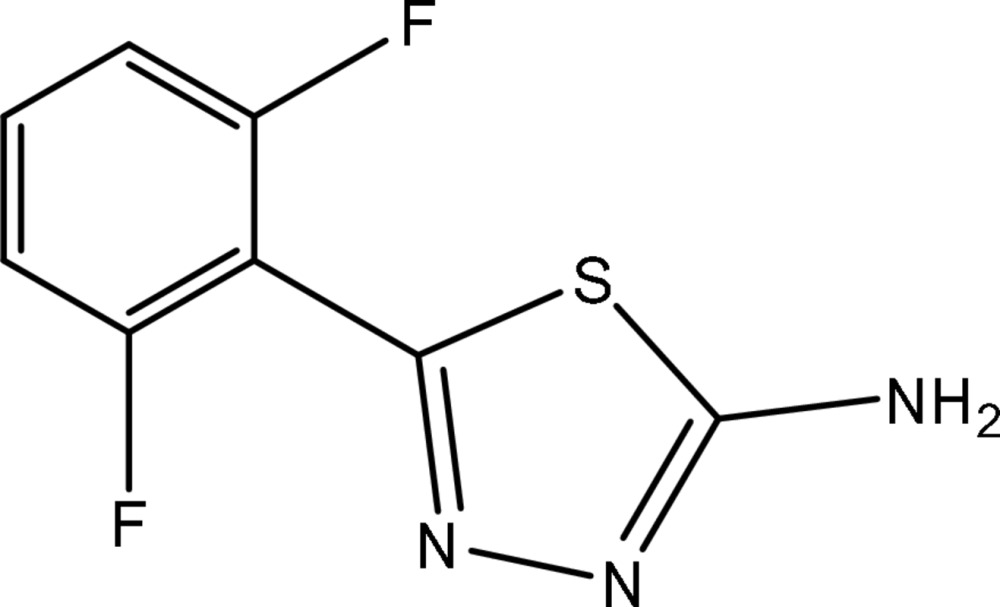


## Experimental

### 

#### Crystal data

C_8_H_5_F_2_N_3_S
*M* *_r_* = 213.21
Monoclinic, 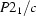

*a* = 9.0920 (18) Å
*b* = 8.7400 (17) Å
*c* = 10.936 (2) Å
β = 95.85 (3)°
*V* = 864.5 (3) Å^3^
*Z* = 4
Mo *K*α radiation
μ = 0.37 mm^−1^
*T* = 293 K
0.20 × 0.10 × 0.10 mm

#### Data collection

Enraf–Nonius CAD-4 diffractometer
Absorption correction: ψ scan (North *et al.*, 1968 ▶) *T*_min_ = 0.931, *T*_max_ = 0.964
1670 measured reflections
1568 independent reflections
1189 reflections with *I* > 2σ(*I*)
*R*_int_ = 0.018
3 standard reflections every 200 reflections intensity decay: 1%

#### Refinement

*R*[*F* ^2^ > 2σ(*F* ^2^)] = 0.042
*wR*(*F* ^2^) = 0.109
*S* = 1.01
1568 reflections
127 parameters
H-atom parameters constrained
Δρ_max_ = 0.26 e Å^−3^
Δρ_min_ = −0.28 e Å^−3^

### 

Data collection: *CAD-4 EXPRESS* (Enraf–Nonius, 1989 ▶); cell refinement: *CAD-4 EXPRESS*; data reduction: *XCAD4* (Harms & Wocadlo, 1995 ▶); program(s) used to solve structure: *SHELXS97* (Sheldrick, 2008 ▶); program(s) used to refine structure: *SHELXL97* (Sheldrick, 2008 ▶); molecular graphics: *SHELXTL* (Sheldrick, 2008 ▶); software used to prepare material for publication: *SHELXL97*.

## Supplementary Material

Crystal structure: contains datablocks global, I. DOI: 10.1107/S1600536809047990/rn2058sup1.cif

Structure factors: contains datablocks I. DOI: 10.1107/S1600536809047990/rn2058Isup2.hkl

Additional supplementary materials:  crystallographic information; 3D view; checkCIF report

## Figures and Tables

**Table 1:** Hydrogen-bond geometry (Å, °)

*D*—H⋯*A*	*D*—H	H⋯*A*	*D*⋯*A*	*D*—H⋯*A*
N3—H3*A*⋯N2^i^	0.86	2.17	3.017 (4)	166
N3—H3*B*⋯N1^ii^	0.86	2.30	3.088 (3)	152

Symmetry codes: (i) 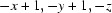; (ii) 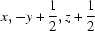.

**Fig. 1.**
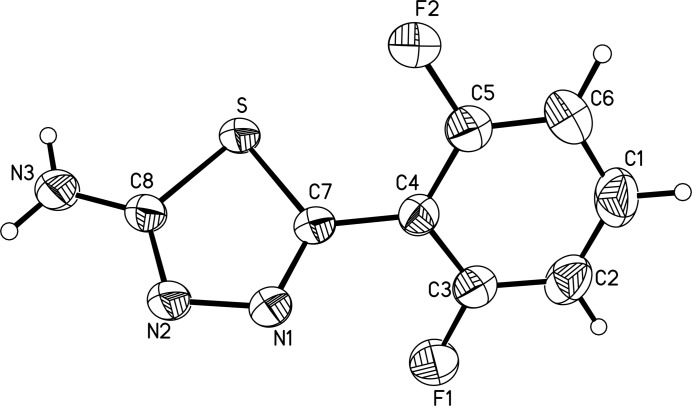
A view of the molecular structure of (I). Displacement ellipsoids are drawn at the 50% probability level.

**Fig. 2.**
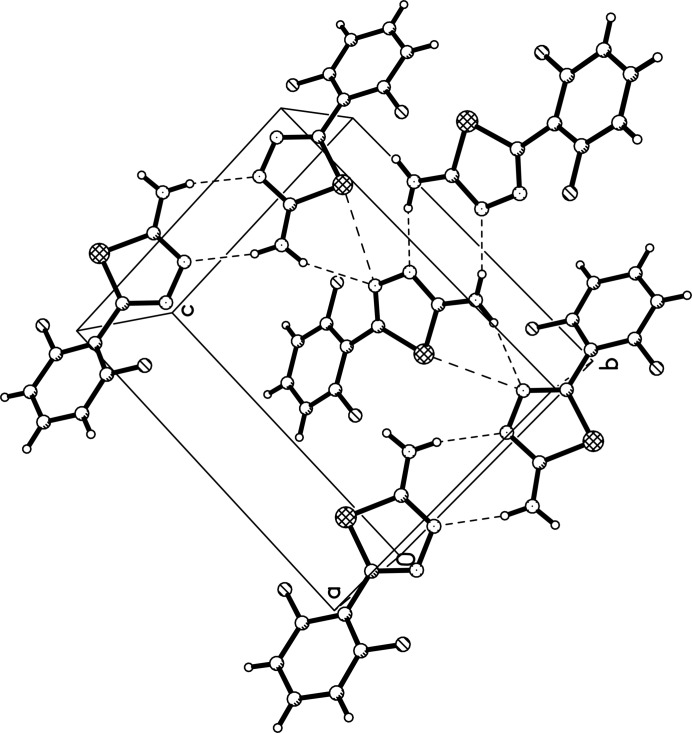
Partial packing view showing the hydrogen-bonded network. Dashed lines indicate intermolecular N—H···N hydrogen bonds and intermolecular N-H···S contacts between the molecules.

## supplementary crystallographic information

### Comment

1,3,4-Thiadiazole derivatives represent a class of biologically important compounds, which often exhibit insecticidal, fungicidal and other biological activities (Nakagawa *et al.*, 1996; Wang *et al.*, 1999). We report here the crystal structure of the title compound, (I).

The molecular structure of (I) is shown in Fig.1, in which the bond lengths and angles are generally within normal ranges (Allen *et al.*, 1987). The dihedral angle between the thiadiazole and phenyl ring is 35.19 (14)°. In the crystal structure, intermolecular N—H···N hydrogen bonds (Fig. 2) form chains along the b and c axes. There are also intermolecular N-H···S contacts between the molecules, which may further stabilize the structure.

### Experimental

2,6-difluorobenzoic acid (2 mmol) and thiosemicarbazide (5 mmol) were mixed in a 25 ml flask, and kept in the oil bath at 90°C for 6 h. After cooling, the crude product (I) precipitated and was filtrated. Pure compound (I) was obtained by crystallization from ethanol (20 ml). Crystals of (I) suitable for X-ray diffraction were obtained by slow evaporation of an acetone solution.

### Refinement

All H atoms bonded to the C atoms were placed geometrically at distances of 0.93–0.97 Å and included in the refinement in riding motion approximation with *U*_iso_(H) = 1.2 or 1.5*U*_eq_ of the carrier atom.

### Crystal data

**Table:**

C_8_H_5_F_2_N_3_S	*D*_x_ = 1.638 Mg m^−^^3^
*M**_r_* = 213.21	Melting point: 533 K
Monoclinic, *P*2_1_/*c*	Mo *K*α radiation, λ = 0.71073 Å
*a* = 9.0920 (18) Å	Cell parameters from 25 reflections
*b* = 8.7400 (17) Å	θ = 10–13°
*c* = 10.936 (2) Å	µ = 0.37 mm^−^^1^
β = 95.85 (3)°	*T* = 293 K
*V* = 864.5 (3) Å^3^	Block, colorless
*Z* = 4	0.20 × 0.10 × 0.10 mm
*F*(000) = 432	

### Data collection

**Table:**

Enraf–Nonius CAD-4 diffractometer	1189 reflections with *I* > 2σ(*I*)
Radiation source: fine-focus sealed tube	*R*_int_ = 0.018
graphite	θ_max_ = 25.3°, θ_min_ = 2.3°
ω/2θ scans	*h* = 0→10
Absorption correction: ψ scan (North *et al.*, 1968)	*k* = 0→10
*T*_min_ = 0.931, *T*_max_ = 0.964	*l* = −13→13
1670 measured reflections	3 standard reflections every 200 reflections
1568 independent reflections	intensity decay: 1%

### Refinement

**Table:**

Refinement on *F*^2^	Primary atom site location: structure-invariant direct methods
Least-squares matrix: full	Secondary atom site location: difference Fourier map
*R*[*F*^2^ > 2σ(*F*^2^)] = 0.042	Hydrogen site location: inferred from neighbouring sites
*wR*(*F*^2^) = 0.109	H-atom parameters constrained
*S* = 1.01	*w* = 1/[σ^2^(*F*_o_^2^) + (0.060*P*)^2^ + 0.150*P*] where *P* = (*F*_o_^2^ + 2*F*_c_^2^)/3
1568 reflections	(Δ/σ)_max_ < 0.001
127 parameters	Δρ_max_ = 0.26 e Å^−^^3^
0 restraints	Δρ_min_ = −0.28 e Å^−^^3^

### Special details

**Table:**

Geometry. All e.s.d.'s (except the e.s.d. in the dihedral angle between two l.s. planes) are estimated using the full covariance matrix. The cell e.s.d.'s are taken into account individually in the estimation of e.s.d.'s in distances, angles and torsion angles; correlations between e.s.d.'s in cell parameters are only used when they are defined by crystal symmetry. An approximate (isotropic) treatment of cell e.s.d.'s is used for estimating e.s.d.'s involving l.s. planes.
Refinement. Refinement of *F*^2^ against ALL reflections. The weighted *R*-factor *wR* and goodness of fit *S* are based on *F*^2^, conventional *R*-factors *R* are based on *F*, with *F* set to zero for negative *F*^2^. The threshold expression of *F*^2^ > σ(*F*^2^) is used only for calculating *R*-factors(gt) *etc*. and is not relevant to the choice of reflections for refinement. *R*-factors based on *F*^2^ are statistically about twice as large as those based on *F*, and *R*- factors based on ALL data will be even larger.

### Fractional atomic coordinates and isotropic or equivalent isotropic displacement parameters (Å^2^)

**Table:**

	*x*	*y*	*z*	*U*_iso_*/*U*_eq_	
S	0.68075 (8)	0.14488 (8)	0.16016 (6)	0.0423 (2)	
F1	0.8904 (2)	0.0561 (2)	−0.18276 (16)	0.0637 (5)	
N1	0.6608 (3)	0.1922 (3)	−0.07123 (19)	0.0475 (6)	
C1	0.8942 (4)	−0.3350 (4)	−0.0851 (3)	0.0606 (9)	
H1B	0.9319	−0.4285	−0.1078	0.073*	
F2	0.6767 (2)	−0.1812 (2)	0.14183 (15)	0.0616 (5)	
N2	0.6037 (3)	0.3224 (3)	−0.02139 (19)	0.0495 (6)	
C2	0.9208 (3)	−0.2061 (4)	−0.1510 (3)	0.0531 (8)	
H2B	0.9784	−0.2110	−0.2165	0.064*	
N3	0.5574 (3)	0.4251 (3)	0.1678 (2)	0.0521 (7)	
H3A	0.5209	0.5074	0.1338	0.062*	
H3B	0.5620	0.4143	0.2462	0.062*	
C3	0.8608 (3)	−0.0705 (3)	−0.1185 (2)	0.0442 (7)	
C4	0.7736 (3)	−0.0549 (3)	−0.0209 (2)	0.0362 (6)	
C5	0.7556 (3)	−0.1889 (3)	0.0433 (3)	0.0446 (7)	
C6	0.8123 (4)	−0.3281 (3)	0.0144 (3)	0.0571 (8)	
H6A	0.7961	−0.4149	0.0603	0.069*	
C7	0.7062 (3)	0.0912 (3)	0.0101 (2)	0.0357 (6)	
C8	0.6070 (3)	0.3142 (3)	0.0986 (2)	0.0376 (6)	

### Atomic displacement parameters (Å^2^)

**Table:**

	*U*^11^	*U*^22^	*U*^33^	*U*^12^	*U*^13^	*U*^23^
S	0.0628 (5)	0.0382 (4)	0.0272 (3)	0.0092 (3)	0.0101 (3)	0.0052 (3)
F1	0.0799 (13)	0.0567 (11)	0.0601 (11)	0.0030 (10)	0.0351 (10)	0.0104 (9)
N1	0.0737 (17)	0.0411 (12)	0.0278 (11)	0.0138 (12)	0.0064 (11)	−0.0010 (9)
C1	0.069 (2)	0.0476 (18)	0.065 (2)	0.0180 (16)	0.0049 (17)	−0.0093 (16)
F2	0.0825 (13)	0.0493 (10)	0.0580 (11)	0.0069 (9)	0.0309 (10)	0.0111 (8)
N2	0.0803 (18)	0.0405 (13)	0.0280 (11)	0.0176 (12)	0.0069 (11)	0.0017 (10)
C2	0.0503 (18)	0.063 (2)	0.0471 (17)	0.0128 (16)	0.0116 (14)	−0.0065 (15)
N3	0.0824 (19)	0.0448 (13)	0.0304 (12)	0.0185 (13)	0.0128 (12)	0.0008 (10)
C3	0.0479 (16)	0.0468 (16)	0.0384 (14)	0.0020 (13)	0.0071 (13)	0.0000 (12)
C4	0.0389 (15)	0.0363 (14)	0.0333 (13)	0.0015 (11)	0.0035 (11)	−0.0016 (11)
C5	0.0468 (16)	0.0446 (15)	0.0430 (15)	0.0022 (13)	0.0077 (13)	0.0015 (13)
C6	0.069 (2)	0.0377 (16)	0.065 (2)	0.0041 (15)	0.0080 (17)	0.0039 (14)
C7	0.0432 (15)	0.0362 (13)	0.0279 (12)	0.0014 (12)	0.0051 (11)	0.0011 (11)
C8	0.0481 (16)	0.0348 (14)	0.0298 (13)	0.0042 (12)	0.0042 (11)	0.0032 (10)

### Geometric parameters (Å, °)

**Table:**

S—C8	1.733 (3)	C2—C3	1.367 (4)
S—C7	1.745 (2)	C2—H2B	0.9300
F1—C3	1.352 (3)	N3—C8	1.336 (3)
N1—C7	1.291 (3)	N3—H3A	0.8600
N1—N2	1.385 (3)	N3—H3B	0.8600
C1—C2	1.372 (4)	C3—C4	1.400 (4)
C1—C6	1.382 (4)	C4—C5	1.384 (4)
C1—H1B	0.9300	C4—C7	1.471 (3)
F2—C5	1.356 (3)	C5—C6	1.370 (4)
N2—C8	1.311 (3)	C6—H6A	0.9300
			
C8—S—C7	86.98 (12)	C5—C4—C3	114.2 (2)
C7—N1—N2	113.4 (2)	C5—C4—C7	123.0 (2)
C2—C1—C6	121.1 (3)	C3—C4—C7	122.8 (2)
C2—C1—H1B	119.5	F2—C5—C6	118.0 (2)
C6—C1—H1B	119.5	F2—C5—C4	117.4 (2)
C8—N2—N1	112.2 (2)	C6—C5—C4	124.6 (3)
C3—C2—C1	118.6 (3)	C5—C6—C1	117.8 (3)
C3—C2—H2B	120.7	C5—C6—H6A	121.1
C1—C2—H2B	120.7	C1—C6—H6A	121.1
C8—N3—H3A	120.0	N1—C7—C4	123.1 (2)
C8—N3—H3B	120.0	N1—C7—S	113.60 (19)
H3A—N3—H3B	120.0	C4—C7—S	123.26 (18)
F1—C3—C2	117.9 (2)	N2—C8—N3	123.6 (2)
F1—C3—C4	118.4 (2)	N2—C8—S	113.82 (19)
C2—C3—C4	123.7 (3)	N3—C8—S	122.63 (19)
			
C7—N1—N2—C8	−0.5 (4)	C2—C1—C6—C5	1.5 (5)
C6—C1—C2—C3	−1.9 (5)	N2—N1—C7—C4	−178.8 (2)
C1—C2—C3—F1	178.7 (3)	N2—N1—C7—S	0.9 (3)
C1—C2—C3—C4	0.2 (5)	C5—C4—C7—N1	−146.4 (3)
F1—C3—C4—C5	−176.8 (2)	C3—C4—C7—N1	33.2 (4)
C2—C3—C4—C5	1.6 (4)	C5—C4—C7—S	33.9 (4)
F1—C3—C4—C7	3.6 (4)	C3—C4—C7—S	−146.5 (2)
C2—C3—C4—C7	−178.0 (3)	C8—S—C7—N1	−0.7 (2)
C3—C4—C5—F2	177.7 (2)	C8—S—C7—C4	178.9 (2)
C7—C4—C5—F2	−2.7 (4)	N1—N2—C8—N3	−179.6 (3)
C3—C4—C5—C6	−2.1 (4)	N1—N2—C8—S	−0.1 (3)
C7—C4—C5—C6	177.5 (3)	C7—S—C8—N2	0.4 (2)
F2—C5—C6—C1	−179.2 (3)	C7—S—C8—N3	−180.0 (3)
C4—C5—C6—C1	0.6 (5)		

### Hydrogen-bond geometry (Å, °)

**Table:**

*D*—H···*A*	*D*—H	H···*A*	*D*···*A*	*D*—H···*A*
N3—H3A···N2^i^	0.86	2.17	3.017 (4)	166
N3—H3B···N1^ii^	0.86	2.30	3.088 (3)	152

Symmetry codes: (i) −*x*+1, −*y*+1, −*z*; (ii) *x*, −*y*+1/2, *z*+1/2.
